# Genome characterization of a novel binary toxin-positive strain of *Clostridium difficile* and comparison with the epidemic 027 and 078 strains

**DOI:** 10.1186/s13099-017-0191-z

**Published:** 2017-08-07

**Authors:** Zhong Peng, Sidi Liu, Xiujuan Meng, Wan Liang, Zhuofei Xu, Biao Tang, Yuanguo Wang, Juping Duan, Chenchao Fu, Bin Wu, Anhua Wu, Chunhui Li

**Affiliations:** 1 Infection Control Center, Xiangya Hospital of Central South University, Changsha, 410008 Hunan China; 2 State Key Laboratory of Agricultural Microbiology, College of Veterinary Medicine, Huazhong Agricultural University, Wuhan, 430070 Hubei China; 3 MOE Key Laboratory of Animal Genetics, Breeding, and Reproduction, College of Animal Science and Technology, Huazhong Agricultural University, Wuhan, 430070 Hubei China; 4 Institute of Quality and Standard for Agro-products, Zhejiang Academy of Agricultural Sciences, Hangzhou, Zhejiang China; 5 The Hormel Institute, University of Minnesota, Austin, MN 55912 USA; 6 Department of Pharmacy, Changsha Hospital of Traditional Chinese Medicine, Changsha, 410000 Hunan China

**Keywords:** *Clostridium difficile*, ST201, Binary toxin-positive, Whole genome sequencing, Comparative genomic analysis

## Abstract

**Background:**

*Clostridium difficile* is an anaerobic Gram-positive spore-forming gut pathogen that causes antibiotic-associated diarrhea worldwide. A small number of *C. difficile* strains express the binary toxin (CDT), which is generally found in *C. difficile* 027 (ST1) and/or 078 (ST11) in clinic. However, we isolated a binary toxin-positive non-027, non-078 *C. difficile* LC693 that is associated with severe diarrhea in China. The genotype of this strain was determined as ST201. To understand the pathogenesis-basis of *C. difficile* ST201, the strain LC693 was chosen for whole genome sequencing, and its genome sequence was analyzed together with the other two ST201 strains VL-0104 and VL-0391 and compared to the epidemic 027/ST1 and 078/ST11 strains.

**Results:**

The project finally generated an estimated genome size of approximately 4.07 Mbp for strain LC693. Genome size of the three ST201 strains ranged from 4.07 to 4.16 Mb, with an average GC content between 28.5 and 28.9%. Phylogenetic analysis demonstrated that the ST201 strains belonged to clade 3. The ST201 genomes contained more than 40 antibiotic resistance genes and 15 of them were predicted to be associated with vancomycin-resistance. The ST201 strains contained a larger PaLoc with a Tn6218 element inserted than the 027/ST1 and 078/ST11 strains, and encoded a truncated TcdC. In addition, the ST201 strains contained intact binary toxin coding and regulation genes which are highly homologous to the 027/ST1 strain. Genome comparison of the ST201 strains with the epidemic 027 and 078 strain identified 641 genes specific for *C. difficile* ST201, and a number of them were predicted as fitness and virulence associated genes. The presence of those genes also contributes to the pathogenesis of the ST201 strains.

**Conclusions:**

In this study, the genomic characterization of three binary toxin-positive *C. difficile* ST201 strains in clade 3 was discussed and compared to the genomes of the epidemic 027 and the 078 strains. Our analysis identified a number fitness and virulence associated genes/loci in the ST201 genomes that contribute to the pathogenesis of *C. difficile* ST201.

**Electronic supplementary material:**

The online version of this article (doi:10.1186/s13099-017-0191-z) contains supplementary material, which is available to authorized users.

## Background

*Clostridium difficile* infection (CDI) causes huge morbidities and mortalities, as well as great economical burdens throughout the world especially in Europe and North America [1, 2]. Clinical manifestations of CDI range from asymptomatic carriage, to mild or moderate diarrhea, to fulminant colitis [3]. The causative agent of CDI, *C. difficile* is an anaerobic Gram-positive, spore-forming, toxin-producing bacillus that generally colonizes the large intestine of humans and animals [4]. Six distinct phylogenetic clades (clades 1, 2, 3, 4, 5, and C–I) are determined within *C. difficile*, and representatives from most clades are associated with CDI in humans [5]. Prior to 2003, the emergence and prevalence of an epidemic *C. difficile* 027/ST1 with high-level fluoroquinolone resistance in clade 2 and efficient sporulation increases the severity and the harmfulness of CDI [4]. In addition to 027, other recently emerging ribotypes include 001, 017, and 078 [6], and the 078/ST11 strains appear to share the same genetic virulence characteristics as 027 and cause severe disease at a similar rate, but has also been associated with community-acquired infection [7, 8].

Toxin expression is considered as the key contribution factor to the development of CDI [9]. Two main toxins produced by *C. difficile* are TcdA and TcdB, which are generally encoded on a 19.6-kbp pathogenicity locus (PaLoc) [10, 11]. PaLoc also contains another three genes *tcdC*, *tcdE*, and *tcdR* implicated in regulating the expression of the toxins. Besides TcdA and TcdB, approximately 20% of *C. difficle* strains also express the binary toxin (CDT) that is encoded on a locus (CdtLoc) physically separated from the PaLoc [5, 12]. Although the detailed role of CDT in the development of human disease is not well understood, previous data have found that the patients infected with *C. difficile* producing CDT had higher fatality rate (approximately 60%) than those infected with CDT-deficient strains [13]. In clinic, the binary toxin-positive strains are generally 027/ST1 or 078/ST11, and both of them were rarely reported in China [14]. However, we isolated a binary toxin-positive *C. difficile* designated strain LC693 from the fecal sample of a patient with severe diarrhea in China, and the genotype of this strain was neither 027/ST1 nor 078/ST11 but determined as ST201 [14]. To understand the pathogenesis basis of this novel isolate, the strain was then chosen for whole genome sequencing. Comparative genomic analysis of the ST201 strains with the epidemic 027/ST1 strain R20291 and 078/ST11 strain M120 was performed to figure out fitness and virulence associated genes.

## Methods

### Bacterial strains

*Clostridium difficile* ST201 strain LC693 was isolated from the stool specimens from a 65-year-old man with fever, headache, diarrhea, and impaired consciousness. Detailed descriptions of the disease history and clinical diagnose of this man were noted in our previous report [14]. The isolate was determined to be positive for toxin A, toxin B, and binary toxin via PCR assay [15]. In addition to LC693, there are another two ST201 clinical strains whose whole genome sequences are publically available in GenBank: strain VL-0391 (ST201; clinical isolate, recovered date not available, Canada, GenBank Accession No. FALK01000000) and VL-0104 (ST201; clinical isolate, recovered date not available, Canada, GenBank Accession No. FAAJ01000000) [16].

### Genome sequencing, assembly, and annotation

Prior to genomic DNA isolation, a single colony of the strain LC693 was selected from *C. difficile* agar (Sigma, St. Louis, USA) and inoculated in BHIS medium (Brain–heart infusion broth with 10% (w/v) l-cysteine) incubating under an anaerobic atmosphere at 37 °C for 12–24 h. Then the genomic DNA was extracted using QIAGEN Genomic-tip 500/G (QIAGEN, Hilden, Germany) following the manufactory instructions. Total DNA obtained was subjected to quality control by agarose gel electrophoresis and quantified by Qubit (Thermo Fisher Scientific, Waltham, USA). The genome of *C. difficile* L693 was sequenced with massively parallel sequencing (MPS) Illumina technology. A paired-end library with an insert size of 419 bp was sequenced using an Illumina MiSeq by PE300 strategy. Library construction and sequencing were performed at the Beijing Novogene Bioinformatics Technology Co., Ltd (Beijing, China). Quality control of both paired-end and mate-pair reads were performed using in-house program. After this step, Illumina PCR adapter reads and low quality reads were filtered. The filtered reads were assembled by SOAPdenovo [17, 18] to generate contigs. Contigs were then ordered and oriented by mapping them against the reference *C. difficile* 630 genome (GenBank Accession No. NC_009089) using Mauve [19, 20]. Ordered matching contigs were pasted together into a pseudochromosome using a contig linker NNNNNCATTCCATTCATTAATTAATTAATGAATGAATGNNNNN, and nonmatching contigs were tacked on the end in random order, as previous studies did [21, 22]. The LC693 pseudochromosome was then annotated via RAST Server program [23]. Predicted proteins were assigned into the COG database for functional classification [24]. This whole genome shotgun project has been deposited at DDBJ/ENA/GenBank under the Accession NCXL00000000. The version described in this paper is version NCXL01000000. Because there is no annotation information for the genome sequences of strain VL-0391 and VL-0104, therefore, their genome sequences were handled using the same strategy mentioned above.

### Sequence analysis and comparative genomics

Prophages in the genome were predicted by PHAST [25]. Antibiotic resistance-associated genes and virulence-associated genes were determined by performing BLAST analysis of the genome sequence against the antibiotic resistance genes database (ARDB) [26] and the virulence factor database (VFDB) [27], respectively. For comparative analysis, genome sequences of *C. difficile* strains R20291 (027/ST1, recent epidemic and hypervirulent, clade 2) and M120 (078/ST11, hypervirulent, clade 5) as well as their annotations were retrieved from GenBank under Accession Numbers FN545816 and NC_017174, respectively. Sequence comparisons were performed using either BRIG software [28], progressive-Mauve procedure [29], or Easyfig software [30]. Single nucleotide polymorphisms (SNPs) between *C. difficile* genomes were also exported via progressive-Mauve [29]. The coding effects of SNPs were analyzed using a local Perl command reported before [31]. Orthologous proteins were differentiated via BLUSTCLUST (version 2.2.24) for amino acids with the identity ≥90% plus alignment coverage ≥90% and an e-value of 1e-6 as cut-off. Phylogenetic tree was constructed and graphically presented by MEGA 7.0 [32] based on the sequences of seven conserved house-keeping genes *adk*, *atpA*, *dx*, *glyA*, *recA*, *sodA*, and *tpi*, using neighbor-joining algorithm with 1000 bootstrapping.

## Results

### Phylogeny

**Fig. 1:**
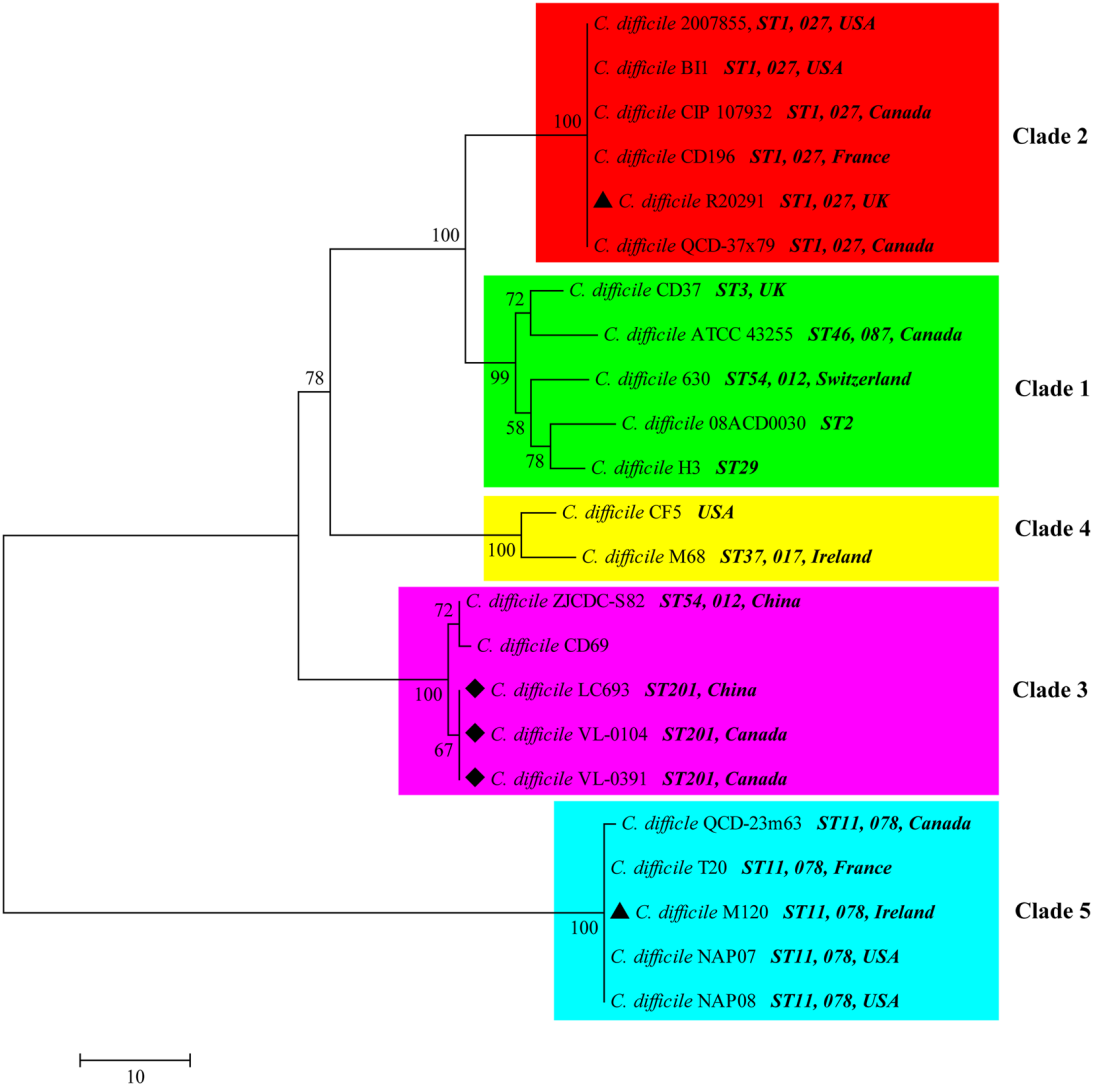
Evolutionary relationships of *Clostridium difficile* clinical strains. The evolutionary history was inferred using the neighbor-joining method. The optimal tree with the sum of branch length = 182.92187500 is shown. The percentage of replicate trees in which the associated taxa clustered together in the bootstrap test (1000 replicates) are shown next to the branches. The tree is drawn to scale, with branch lengths in the same units as those of the evolutionary distances used to infer the phylogenetic tree. The evolutionary distances were computed using the number of differences method and are in the units of the number of base differences per sequence. The analysis involved 23 nucleotide sequences. All positions containing gaps and missing data were eliminated. There were a total of 7050 positions in the final dataset. Evolutionary analyses were conducted in MEGA7

Phylogenetic analysis based on conserved genes across the *C. difficile* genomes showed that the five *C. difficile* clinical isolates discussed in this study belonged to three different clades (Fig. 1). All ST201 strains were members of clade 3, while the epidemic 027/ST1 strain R20291 and 078/ST11 strain M120 belonged to clade 2 and clade 5, respectively. More interestingly, all 027/ST1 clinical strains were concentrated in clade 2 and the 078/ST11 strains were included in clade 5 (Fig. 1).

### Overview of the *C. difficile* ST201 genomes

Whole genome sequencing strategy on *C. difficile* strain LC693 yielded a total of 1,413,333 reads with 106-fold coverage (Q20 98.43%, Q30 94.48%). Those reads were then used to the draft assemble, generating 146 contigs larger than 500 bp, of which the largest one was 150,334 bp in length. The contigs were then mapped to *C. difficile* 630 genome sequence to generate an estimated genome size of approximately 4.07 Mbp. This size was quite similar to another ST201 strain VL-0104, but was approximately 8.8 kb smaller than strain VL-0391 (Table 1). Genome sizes of the ST201 strains were located between the genome of the 078/ST11 strain M120 and the 027/ST1 strain R20291. The average GC contents of the ST201 genome sequences were also near, between 28.5 and 28.9%. Those contents were also similar to the 027/ST1 and 078/ST11 genome sequences. No plasmids were identified in the genome sequences discussed in this study (Table 1). According to annotation using RAST Server, the ST201 genomes carried 3921–3956 predicted open reading frames (ORFs), which corresponded to 3868–3833 putative coding DNA sequences (CDSs), 69–79 tRNAs and 9–41 rRNAs (Table 1).

**Table 1:** General features of the *C. difficile* genomes

Strain	LC693	VL-0104	VL-0391	R20291	M120
Places of isolation	China	Canada	Canada	UK	UK
Ribotype	–	–	–	027	078
Sequence type	ST201	ST201	ST201	ST1	ST11
Toxin profile	A + B + CDT+	A + B + CDT+	A + B + CDT+	A + B + CDT+	A + B + CDT+
Genome completion	Draft	Draft	Draft	Complete	Complete
Genome size (bp)	4,073,021	4,068,388	4,160,703	4,191,339	4,047,729
GC%	28.5	28.7	28.9	28.6	28.7
Predicted CDSs	3868	3833	3832	3508	3490
Predicted tRNAs	79	77	69	65	86
Predicted rRNAs	9	11	41	28	32
Plasmid	0	0	0	0	0
Prophages	7 (3^a^)	7 (2^a^)	8 (3^a^)	4 (2^a^)	4 (1^a^)

^a^Indicates the number of intact prophages

### Antibiotic resistance associated genes

The antibiotic resistance proteins were figured out by performing BLAST analysis of the CDSs predicted in the ST201 genomes against the ARDB database using a percent identity over 40% and an E value of 10^−4^. The prediction identified 40 (LC693 and VL-0104) to 41 (VL-0391) putative antibiotic resistance associated genes within the ST201 genomes (Table 2). Based on their functional predictions, a total of 15 genes conferred vancomycin-resistance to the three ST201 strains (nine mediated vancomycin-resistance only and another six mediated both vancomycin- and teicoplanin-resistance); 11 (LC693 and VL-0104) or 12 genes (VL-0391) mediated macrolide-resistance; the rest conferred resistance to other antibiotics to the ST201 strains: bacitracin (7 genes), streptogramin A (4 genes), deoxycholate (1 gene), fosfomycin (1 gene), tetracycline (1 gene) and fluoroquinolone (1 gene). It is worthy of note that broth microdilution test showed that the minimum inhibitory concentration of vancomycin to strain LC693 was 4 μg/ml. This result suggests that strain LC693 is resistant to vancomycin, according to EUCAST breakpoint (http://www.eucast.org/clinical_breakpoints/). Interestingly, all antibiotic resistance genes identified LC693 were homologous to those predicted in VL-0104 genome, and 39 of them were also homologous to those determined in VL-0391, with the exception of a vancomycin-resistance-associated gene (Table 2). In addition, most of the antibiotic resistance genes determined in the ST201 genomes were also found in the ST1 and ST11 genomes (Table 2).

**Table 2:** Antibiotic resistance associated proteins predicted in the ST201 genomes

Locus in LC693	Locus in VL-0104	Locus in VL-0391	Length (aa)	Description	Resistance	Presence in R20291	Presence in M120
0042	2926	1001	196	Virginiamycin A acetyltransferase, which can inactivate the target drug	Streptogramin_a	+	+
0115	2999	1075	197	Virginiamycin A acetyltransferase, which can inactivate the target drug	Streptogramin_a	+	−
0120	3004	1080	241	VanA type vancomycin resistance operon genes, which can synthesize peptidoglycan with modified C-terminal d-Ala-d-Ala to d-alanine-d-lactate	Teicoplanin; vancomycin	+	+
0267	3195	1228	65	Major facilitator superfamily transporter. Multidrug resistance efflux pump	Deoxycholate; fosfomycin	−	+
0402	3309	1347	228	Resistance-nodulation-cell division transporter system. Multidrug resistance efflux pump. Macrolide-specific efflux system	Macrolide	+	+
0410	3317	1355	228	Resistance-nodulation-cell division transporter system. Multidrug resistance efflux pump. Macrolide-specific efflux system	Macrolide	+	+
0412	3319	1357	223	VanA type vancomycin resistance operon genes, which can synthesize peptidoglycan with modified C-terminal d-Ala-d-Ala to d-alanine-d-lactate	Teicoplanin; vancomycin	+	+
0416	3323	1361	221	Resistance-nodulation-cell division transporter system. Multidrug resistance efflux pump. Macrolide-specific efflux system	Macrolide1094	+	+
0576	3486	1519	222	VanB type vancomycin resistance operon genes, which can synthesize peptidoglycan with modified C-terminal d-Ala-d-Ala to d-alanine-d-lactate	Vancomycin	+	+
0580	3490	1523	607	Ribosomal protection protein, which protects ribosome from the translation inhibition of tetracycline	Tetracycline	+	+
0601	3511	1544	67	VanG type vancomycin resistance operon genes, which can synthesize peptidoglycan with modified C-terminal d-Ala-d-Ala to d-alanine-d-serine	Vancomycin	+	+
0699	3617	1658	210	Virginiamycin A acetyltransferase, which can inactivate the target drug	Streptogramin_a	+	+
0805	3681	1722	230	VanG type vancomycin resistance operon genes, which can synthesize peptidoglycan with modified C-terminal d-Ala-d-Ala to d-alanine-d-serine	Vancomycin	+	+
0820	3697	1738	238	VanG type vancomycin resistance operon genes, which can synthesize peptidoglycan with modified C-terminal d-Ala-d-Ala to d-alanine-d-serine	Vancomycin	+	+
1054	0094	1967	227	Resistance-nodulation-cell division transporter system. Multidrug resistance efflux pump. Macrolide-specific efflux system	Macrolide	+	+
1094	0134	2007	238	VanG type vancomycin resistance operon genes, which can synthesize peptidoglycan with modified C-terminal d-Ala-d-Ala to d-alanine-d-serine	Vancomycin	+	+
1247	0352	2230	234	VanA type vancomycin resistance operon genes, which can synthesize peptidoglycan with modified C-terminal d-Ala-d-Ala to d-alanine-d-lactate	Teicoplanin; vancomycin	+	+
1500	0582	2467	283	Undecaprenyl pyrophosphate phosphatase, which consists in the sequestration of Undecaprenyl pyrophosphate	Bacitracin	+	+
1568	0651	2535	250	Resistance-nodulation-cell division transporter system. Multidrug resistance efflux pump. Macrolide-specific efflux system	Macrolide	+	+
1685	0767	−	238	VanG type vancomycin resistance operon genes, which can synthesize peptidoglycan with modified C-terminal d-Ala-d-Ala to d-alanine-d-serine	Vancomycin	−	−
1799	0854	2700	110	ABC transporter system, bacitracin efflux pump	Bacitracin	−	−
1869	0925	3336	371	VanA type vancomycin resistance operon genes, which can synthesize peptidoglycan with modified C-terminal d-Ala-d-Ala to d-alanine-d-lactate	Teicoplanin; vancomycin	+	−
1870	0926	3337	232	VanA type vancomycin resistance operon genes, which can synthesize peptidoglycan with modified C-terminal d-Ala-d-Ala to d-alanine-d-lactate	Teicoplanin; vancomycin	+	−
1962	1044	2900	276	VanG type vancomycin resistance operon genes, which can synthesize peptidoglycan with modified C-terminal d-Ala-d-Ala to d-alanine-d-serine	Vancomycin	+	−
2234	1252	3136	228	Resistance-nodulation-cell division transporter system. Multidrug resistance efflux pump. Macrolide-specific efflux system	Macrolide	+	+
2251	1268	3166	230	VanG type vancomycin resistance operon genes, which can synthesize peptidoglycan with modified C-terminal d-Ala-d-Ala to d-alanine-d-serine	Vancomycin	+	+
2272	1289	3187	288	Pentapeptide repeat family, which protects DNA gyrase from the inhibition of quinolones	Fluoroquinolone	+	+
2466	1548	3530	305	ABC transporter system, bacitracin efflux pump	Bacitracin	+	+
2642	1673	3662	227	Resistance-nodulation-cell division transporter system. Multidrug resistance efflux pump. Macrolide-specific efflux system	Macrolide	+	+
2655	1998	3676	234	ABC transporter system, bacitracin efflux pump	Bacitracin	+	+
2658	1995	3679	230	VanA type vancomycin resistance operon genes, which can synthesize peptidoglycan with modified C-terminal d-Ala-d-Ala to d-alanine-d-lactate	Teicoplanin; vancomycin	+	+
2661	1992	3682	222	Resistance-nodulation-cell division transporter system. Multidrug resistance efflux pump. Macrolide-specific efflux system	Macrolide	+	+
2663	1990	3684	224	VanE type vancomycin resistance operon genes, which can synthesize peptidoglycan with modified C-terminal d-Ala-d-Ala to d-alanine-d-serine	Vancomycin	+	+
2831	1853	0043	304	ABC transporter system, bacitracin efflux pump	Bacitracin	+	+
2922	2024	0104	192	Virginiamycin A acetyltransferase, which can inactivate the target drug	Streptogramin_a	−	+
3194	2245	0335	227	Resistance-nodulation-cell division transporter system. Multidrug resistance efflux pump. Macrolide-specific efflux system	Macrolide	+	+
3599	2639	0706	274	Undecaprenyl pyrophosphate phosphatase, which consists in the sequestration of Undecaprenyl pyrophosphate	Bacitracin	+	+
3614	2654	0729	235	ABC transporter system, bacitracin efflux pump	Bacitracin	+	+
3781	2789	0865	225	Resistance-nodulation-cell division transporter system. Multidrug resistance efflux pump. Macrolide-specific efflux system	Macrolide	+	+
3846	2855	0930	224	Resistance-nodulation-cell division transporter system. Multidrug resistance efflux pump. Macrolide-specific efflux system	Macrolide	+	+
−	−	1192	71	Resistance-nodulation-cell division transporter system. Multidrug resistance efflux pump. Macrolide-specific efflux system	Macrolide	−	−
−	−	3284	174	VanG type vancomycin resistance operon genes, which can synthesize peptidoglycan with modified C-terminal d-Ala-d-Ala to d-alanine–d-serine	Vancomycin	−	−

“+” indicates corresponding genes present; “−” indicates no corresponding genes

### Prophage identification

Based on the prediction by PHAST, the ST201 genome sequences contained seven to eight prophages (Table 3). Strain LC693 contained three intact, three incomplete and one questionable phages. Among those prophages, three prophages (the 19.7-kb, the 71.7-kb and the 67.2-kb one) were also present in the other two ST201 strains, but they were missing in the ST1 strain and the ST11 strain. Another 27.1-kb prophage was not only shared by the other two ST201 isolates but also shared by the ST1 and ST11 strains. Moreover, the homologous region (97–98% identity; 82–99% coverage) of this putative phage was also found in genomes of *C. difficile* strains of other clades such as strains 630 and 08ACD0030 (clade 1), strains M68 and CF5 (clade 4).

**Table 3:** Prophages predicted in the three ST201 genomes

Phage region	Region length (kb)	Completeness	#CDS	Possible phage	GC (%)
Strain LC693					
1	28.3	Incomplete	24	PHAGE_Clostr_phiCD505_NC_028764 (6)	27.38
2	19.7	Incomplete	47	PHAGE_Clostr_CDMH1_NC_024144 (21)	28.15
3	71.7	Intact	102	PHAGE_Clostr_phiCDHM19_NC_028996 (35)	29.33
4	27.1	Incomplete	31	PHAGE_Clostr_phiCDHM19_NC_028996 (11)	28.04
5	7.4	Questionable	6	PHAGE_Paenib_Xenia_NC_028837 (2)	30.32
6	67.2	Intact	88	PHAGE_Clostr_phiMMP02_NC_019421 (30)	28.69
7	24.1	Intact	45	PHAGE_Clostr_c_st_NC_007581 (6)	33.26
Strain VL-0104					
1	22.4	Incomplete	19	PHAGE_Clostr_c_st_NC_007581 (3)	28.77
2	48.3	Intact	58	PHAGE_Clostr_CDMH1_NC_024144 (22)	28.44
3	27.1	Incomplete	31	PHAGE_Clostr_phiCDHM19_NC_028996 (11)	28.04
4	48.4	Intact	69	PHAGE_Clostr_phiCD505_NC_028764 (24)	28.27
5	7.4	Questionable	6	PHAGE_Paenib_Xenia_NC_028837 (2)	30.32
6	15.6	Incomplete	27	PHAGE_Clostr_phiCD27_NC_011398 (14)	29.98
7	11.2	Questionable	18	PHAGE_Clostr_c_st_NC_007581 (4)	31.68
Strain VL-0391					
1	22.6	Incomplete	18	PHAGE_Clostr_c_st_NC_007581 (3)	28.79
2	33.3	Intact	39	PHAGE_Clostr_phiMMP03_NC_028959 (8)	27.45
3	27.1	Incomplete	31	PHAGE_Clostr_phiCDHM19_NC_028996 (11)	28.04
4	51.4	Intact	69	PHAGE_Clostr_phiCD505_NC_028764 (23)	28.06
5	7.4	Questionable	6	PHAGE_Paenib_Xenia_NC_028837 (2)	30.32
6	34.9	Questionable	37	PHAGE_Clostr_CDMH1_NC_024144 (15)	31.25
7	25.3	Incomplete	34	PHAGE_Clostr_phiCD27_NC_011398 (12)	31.52
8	11.1	Intact	23	PHAGE_Clostr_c_st_NC_007581 (3)	32.47

### Single nucleotide polymorphisms

Single nucleotide polymorphisms analysis showed that the ST201 genomes harbored approximately 53,288 SNPs (52,447–54,837) and 107,774 SNPs (107,694–107,889) compared to the ST1 genome and the ST11 genome, respectively (Table 4). Among them, approximately 40,224 (39,065–41,696) and 82,383 (81,424–82,872) SNPs were found in the coding sequence regions across the ST201 genomes, and 14,662 (14,127–15,172) and 25,649 (25,046–26,258) of those SNPs caused non-synonymous changes, respectively. The average ratio of nonsynonymous versus synonymous substitution rate (dN/dS) of the SNPs identified the ST201 genomes against the ST1 genome was 0.57, and 0.45 against the ST11 genome.

**Table 4:** SNPs identified in the ST201 genomes against the ST1 and the ST11 genomes

	Nos. of SNPs	Non-synonymous	Synonymous	DN/dS
LC693 vs. R20291	54,837 (41,696^a^)	15,172	26,524	0.57
VL-0104 vs. R20291	52,580 (39,912^a^)	14,687	25,225	0.58
VL-0319 vs. R20291	52,447 (39,065^a^)	14,127	24,938	0.57
Subtotal	159,864 (120,673^a^)	43,986	76,687	0.57
LC693 vs. M120	107,738 (82,855^a^)	25,643	57,212	0.45
VL-0104 vs. M120	107,889 (82,872^a^)	26,258	56,614	0.46
VL-0319 vs. M120	107,694 (81,424^a^)	25,046	56,378	0.44
Subtotal	323,321 (247,151^a^)	76,947	170,204	0.45

^a^Indicates the number of SNPs in CDSs

### Sequence analysis of PaLoc

**Fig. 2:**
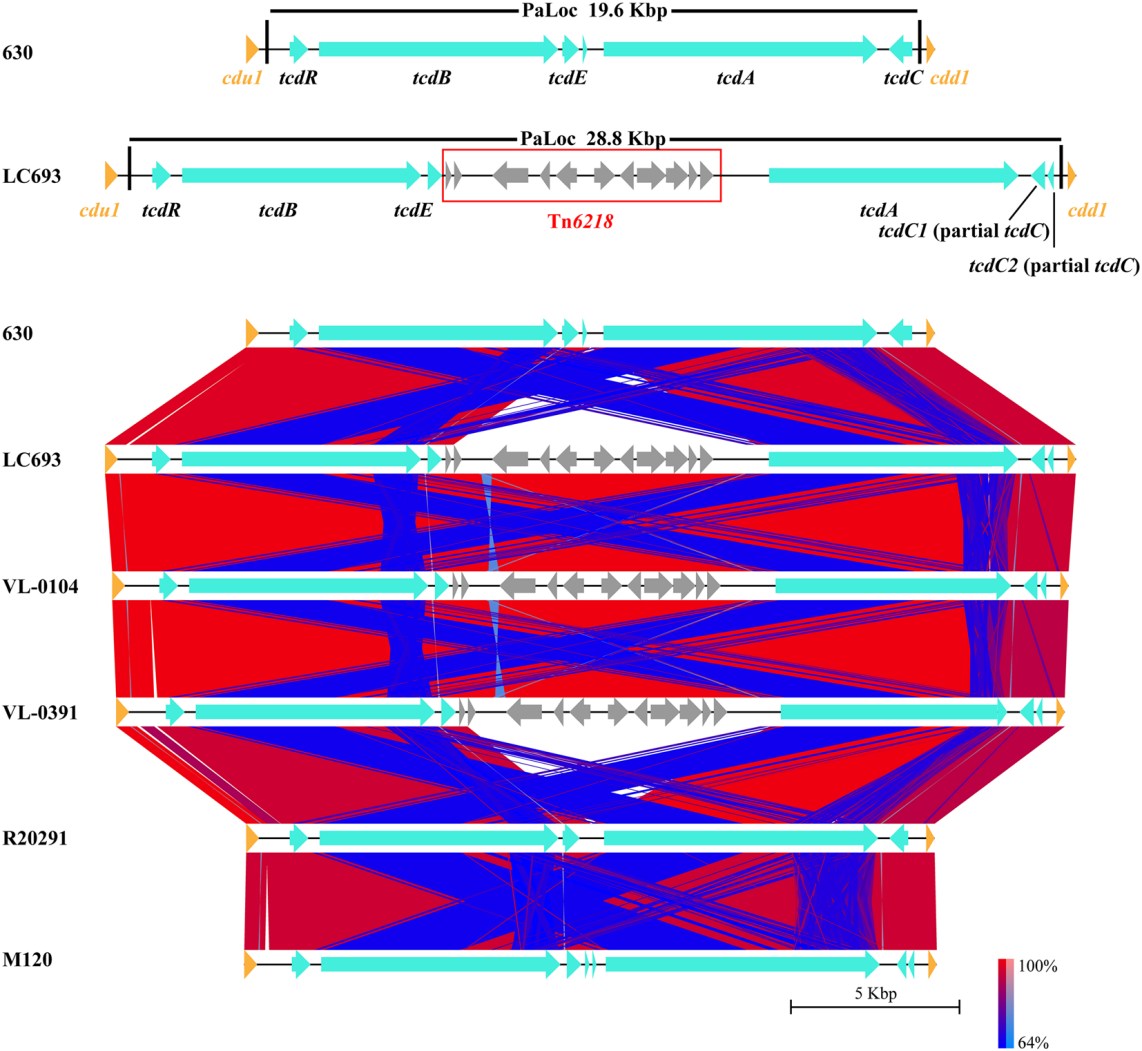
Comparative analysis of PaLoc *Clostridium difficile* strains discussed in this study. *Color* code stands for BLASTn identity of those regions between genomes *Arrows* in the same colors represent putative CDSs with similar roles in different genomes

Our previous study has determined that the ST201 strain LC693 was TcdA- and TcdB-positive [14], and the two large clotridial toxins TcdA and TcdB are reported to be encoded on the 19.6-kb PaLoc between two conserved genes designed *cdd1* and *cdu1* [5, 10, 11]. However, the PaLoc region carried by the three ST201 strains discussed here was found to be located in a 28.8-kb region, with a specific fragment of approximately 9-kb in length inserted between the putative *tcdE* gene and the *tcdA* gene that was missing in the 19.6-kb PaLoc contained by the epidemic ST1 strain R20291 and ST11 strain M120 (Fig. 2). Interestingly, this 9-kb insertion was also found in the ST54 strain ZJCDC-S82, and it contained approximately 10 predicted genes. Nucleotide sequence comparison using BlastN against the NCBI nucleotide collection database found that this this 9-kb insertion was highly homologous (99% nucleotide sequence identity) to the novel mobile genetic element Tn*6218* identified in the PaLocs of clade 3 strains [33]. Correspondingly, orthologs of the four common genes (*int*, *xis*, *rep*, and *xre*) and five accessory genes (a transcription regulator gene *merR*; a gene encoding the oxidoreductase; the flavodoxin coding gene; an orf encoding a hypothetical protein containing the cupin domain; and the RNA polymerase σ70 coding gene) carried by Tn*6218* determined before [33] were expectedly found in the 9-kb insertion contained by the ST201 strains.

The TcdA and TcdB encoding genes *tcdA* and *tcdB* harbored by the ST201 strains were highly homologous to that carried by the ST1 strain or the ST11 strain. Moreover, those two genes were more conserved among the strains in the same clade other than among those in different clades (Table 5). In addition, the SNPs identified with either the *tcdA* gene and/or the *tcdB* gene between the ST201 strains and the ST11 strain were much less than those between the ST201 strains and the ST1 strain (Table 5).

**Table 5:** SNPs harbored by the PaLoc comprising genes of the ST201 strains compared with isolates of ST1 and ST11

Strains	*tcdR*	*tcdB*	*tcdE*	Tn*6218*									*tcdA*	*tcdC*
				*tnt*	*xis*	*rep*	*xre*	*merR*	Oxidoreductase	Flavodoxin	orf	σ70		
Against R20291 (ST1/027, clade 2)														
LC693	13	459	3 (72 del)	–	–	–	–	–	–	–	–	–	100	5 (144 del)
VL-0104	13	459	3 (72 del)	–	–	–	–	–	–	–	–	–	100	5 (144 del)
VL-0391	13	459	3 (72 del)	–	–	–	–	–	–	–	–	–	100	5 (144 del)
Against M120 (ST11/078, clade 5)														
LC693	18	175	2 (72 del)	–	–	–	–	–	–	–	–	–	90	3 (264 del + 20 in)
VL-0104	18	175	2 (72 del)	–	–	–	–	–	–	–	–	–	90	3 (264 del + 20 in)
VL-0391	18	175	2 (72 del)	–	–	–	–	–	–	–	–	–	90	3 (264 del + 20 in)

**Fig. 3:**
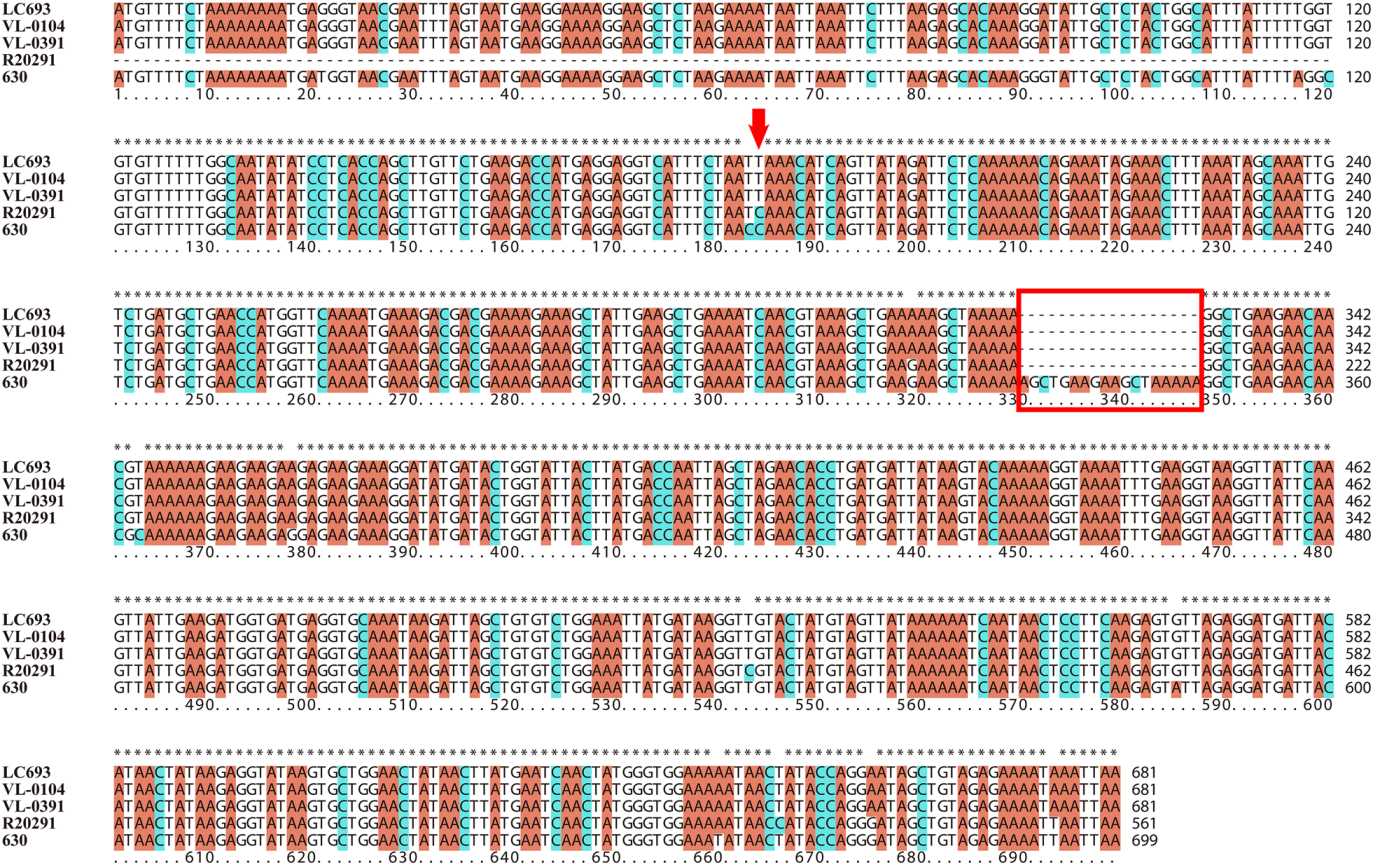
Sequence comparisons of *tcdC* among *Clostridium difficile* strains discussed in this study

**Fig. 4:**
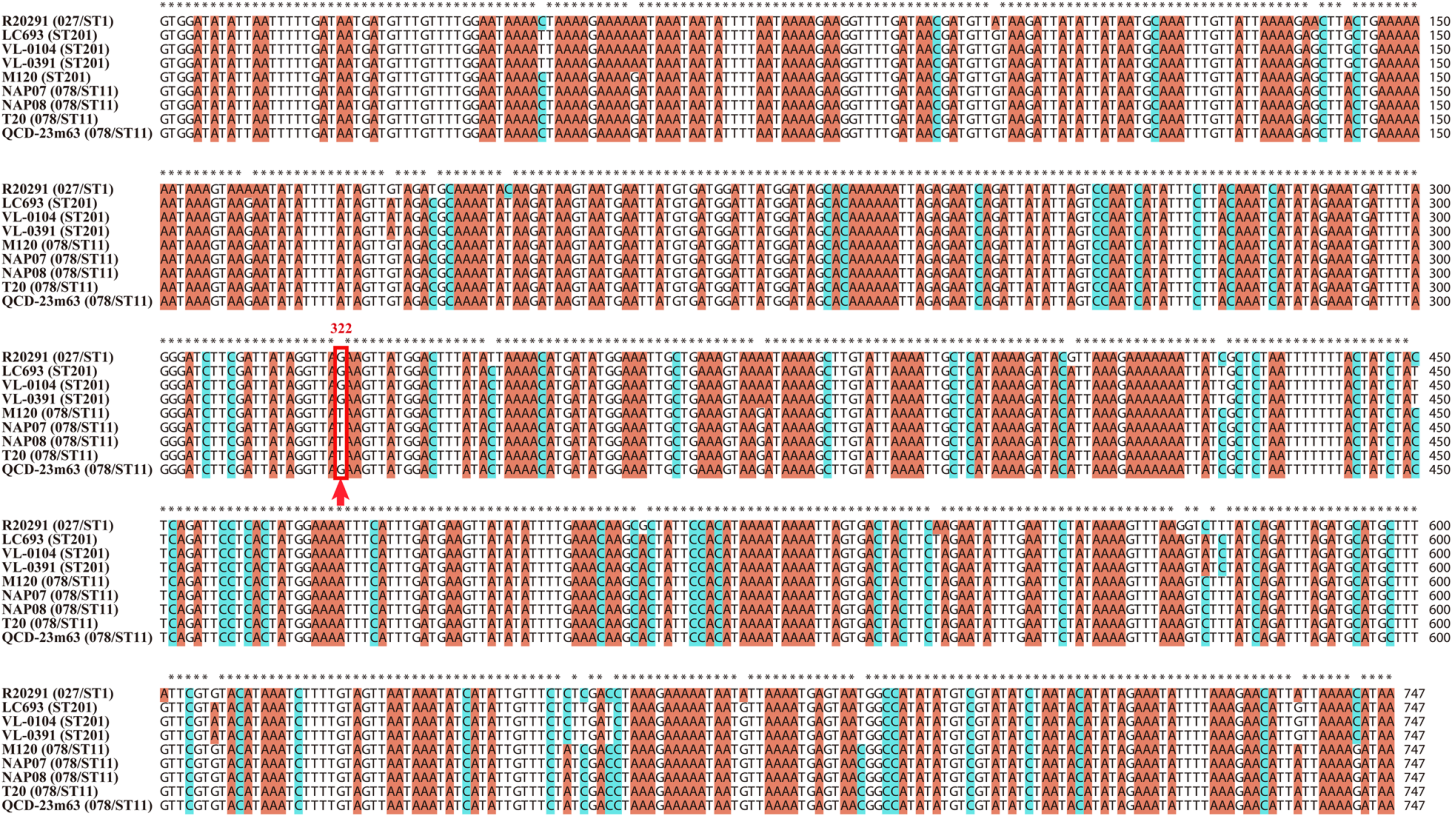
Sequence comparisons of *cdtR* among *Clostridium difficile* strains discussed in this study

Among the toxin-expression regulating genes, *tcdR* was also conserved, as only 13 SNPs (between ST201 and ST1) and 18 SNPs (between ST201 and ST11) were identified between different clade strains. However, more variations were observed within the *tcdE* gene and the *tcdC* gene among the strains in different clades. The *tcdE* gene carried by the three ST201 strains in clade 3 had a 72-bp deletion at the N-terminal of the gene compared to the ST1 strain in clade 2 and/or the ST11 strain in clade 5 (Fig. 2; Table 5). However, for the *tcdC* gene, it was very interesting that there were two potential genes in the putative *tcdC* region of the ST201 genomes as well as in M120 compared to strain R20291 (Fig. 2). Further analysis using the putative *tcdC* region of strain LC693 comparing with the typical *tcdC* nucleotide sequence of strain 630 found a nucleotide change occurred at position 185 (C → T) which caused the formation of a stop codon here and led to an early termination of translation and the disruption of the gene (Fig. 4). These mutations resulted in a truncated TcdC protein in the ST201 strains. In addition, an 18-bp deletion was found at positions 330–347 in the putative *tcdC* region of strain LC693 compared to 630 (Fig. 3). Those changed patterns were also found in the other two ST201 strains VL-0104 and VL-0391 (Fig. 3). More interestingly, the *tcdC* harbored by strain R20291 had 120-bp deletion compared to the typical *tcdC* carried by strain 630, and the 18-bp deletion identified in the ST201 genomes at positions 330–347 compared to strain 630 was also found in R20291 (Fig. 3).

### Sequence analysis of CdtLoc

In addition to TcdA and TcdB, the ST201 strain LC693 is also determined as binary-toxin-positive [14]. Sequence comparisons using the nucleotide sequence of the putative CdtLoc locus against the whole genome sequences the other two ST201 strains VL-0104 and VL-0391 as well as the epidemic ST1/027 strain R20291 and the ST11/078 strain M120 demonstrated that the other two ST201 strains also contained the CdtLoc region. Unlike the PaLoc harbored by the clade 3 strains, there were no insertions of mobile genetic elements in the CdtLoc region. Among the three genes carried by CdtLoc, *cdtA* and *ctdB* were highly conserved between the ST201 strains and the ST1/ST11 strains. However, the *ctdR* was found to be conserved among the strains excluding the ST11/078 strain M120. The *cdtR* gene of strain M120 was found to have a nucleotide change occurred at position 322 (G → T) compared to the *ctdR* carried by either the strain R20291 or the three ST201 strains, and this change caused the formation of a stop codon and therefore resulted in a truncated CdtR in M120. Interestingly, this changed pattern was also found in most 078/ST11 strains (Fig. 4).

### Whole genome sequence comparison

**Fig. 5:**
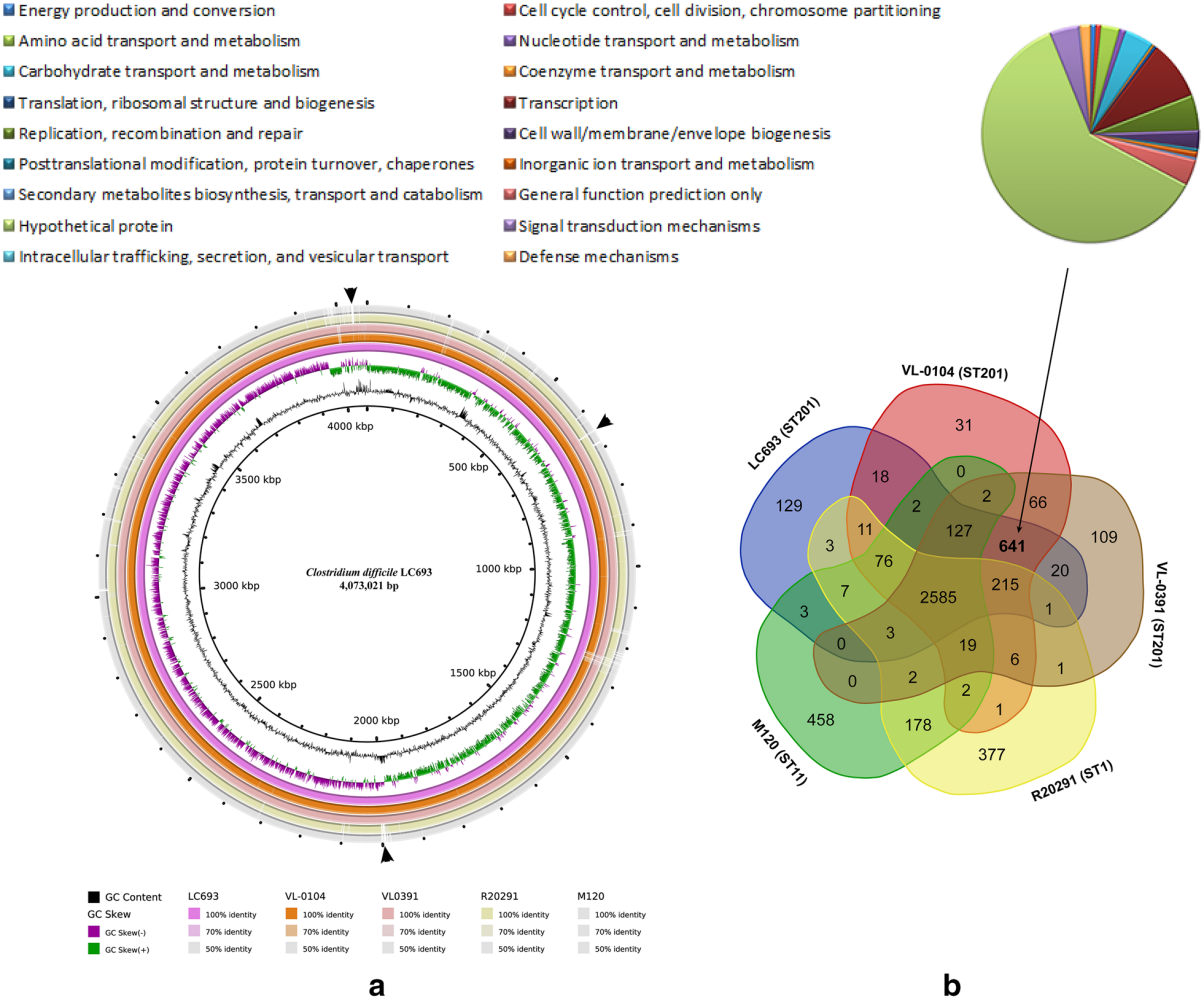
Comparative genomic analysis of *Clostridium difficile* ST201 strains with the epidemic 027/ST1 strain R20291 and 078/ST11 strain M120. **a** Whole genome sequences comparison of the strains. *Circles* from inside to outside indicate GC content of strain LC693, GC skew of strain LC693, *C. difficile* strains LC693, VL-0104, VL-0391, R20291 and M120. Different DNA BLAST identities are shown using different *colors*. **b** Venn diagram shows shared genes and unique gene among the strains. Pie chart displays COG functional catalogues of the 641 predicted genes specific for the ST201 strains

Whole genome sequences comparison showed that the ST201 genomes and the ST1 and the ST11 genomes were highly matched and homologous (Fig. 5a). Comparative analysis identified a shared set of 2585 core genes and a pan genome of more than 1404 genes as well as 31 genes unique to strain VL-0104; 109 unique to VL-0391; 129 unique to LC693; 377 unique to the epidemic ST1/027 strain R20291; and 458 unique to the ST11/078 strain M120 (Fig. 5b). Functional comparison of the core genes and the strain-specific genes against the COG database showed that the core genes mainly participated in carbohydrate transport and metabolism, amino acid transport and metabolism, energy production and conversion, cell membrane biogenesis, inorganic ion transport and metabolism, signal transduction mechanisms, transcription, replication, recombination and repair, coenzyme transport and metabolism, translation, ribosomal structure and biogenesis, nucleotide transport and metabolism, lipid transport and metabolism, posttranslational modification, protein turnover, chaperones, and hypothetical proteins. For the 129 strain-specific genes for LC693, approximately 85 were phage-related genes, and 19, 6, 43, 15, and 2 of them were clustered in the 28.3-, 19.7-, 71.7-, 67.2-, and 24.1-kb prophage that identified in the strain, respectively (Table 3); the rest of them encoded hypothetical proteins, phage-related proteins outside the predicted prophage regions, and proteins in amino acid transport and metabolism, ribosomal structure and biogenesis, transcription, cell membrane biogenesis, inorganic ion transport and metabolism, and defense. The 31 strain-specific genes for VL-0104 encoded proteins mainly participating in cell cycle control, carbohydrate transport and metabolism, transcription, replication, recombination and repair, cell membrane biogenesis, mobilization, and hypothetical proteins. For strain VL-0391, the 109 unique genes encoded proteins associated with energy production and conversion, cell cycle control, amino acid transport and metabolism, carbohydrate transport and metabolism, coenzyme transport and metabolism, lipid transport and metabolism, translation, ribosomal structure and biogenesis, transcription, replication, recombination and repair, cell membrane biogenesis, cell motility, posttranslational modification, inorganic ion transport and metabolism, secondary metabolites biosynthesis, transport and catabolism, signal transduction, intracellular trafficking, secretion, and vesicular transport, and bacterial defense mechanisms.

The three ST201 strains contained 641 genes which were absent in both the ST1 and ST11 strains (Fig. 5b; Additional file 1: Table S1). Those ST201 strains-specific genes contained those predicted as phage-related genes that were carried by either the ST201 strains-shared 19.7-kb, the 71.7-kb or the 67.2-kb prophage. Those ST201 strains-specific genes also included those forming the 9-kb insertion Tn*6218* which was generally found in the clade 3 PaLoc but was absent in other clade strains. In particular, the ST201 strains-specific genes also covered many genes involved in the bacterial fitness and pathogenesis. For example, the type I restriction–modification system was found to have a potential role in the virulence of some bacterial pathogens such as *Haemophilus* [34] and *Salmonella enterica* serovar *Enteritidis* [35]. The ferric iron ABC transporter and the iron compound ABC uptake transporter ATP-binding protein was helpful to uptake iron, which is not only an essential element for bacterial survival, but also acts an environmental signal that regulates the expression of many virulence factors [36]. The histidine kinase and response regulator forms the bacterial two-component system, which is undoubtedly important for bacterial survival and virulence regulation [37]. The antitoxin protein HigA was favorable for bacteria to escape the toxin and was feasible to survival the infection loci [38].

## Discussion

*Clostridium difficile* infection is widely accepted as one of the most common healthcare and economy problems throughout the world especially in North America and Europe [4, 39–41]. More worrisome, the emergence and prevalence of the 027/ST1 has significantly increased the morbidity and mortality of CDI [7, 42, 43]. Besides, the emergent 078/ST11 strains are reported to share the same genetic virulence characteristics as 027/ST1 and cause severe disease at a similar rate [8]. However, both of those two types of strains are rarely reported in China. The 027 has not been detected in China before 2013, and cases of *C. difficile* 078 have not been reported yet [14]. Instead, a number of severe diarrhea-associated *C. difficile* toxigenic strains belonging to clades distinct from the 027/ST1 and 078/ST11 strains have been reported in China [16, 44]. This might indicate that the dominant genotypes of *C. difficile* spreading in China are different from those circulating in North America and Europe. Consistently, phylogenetic analysis showed that the novel binary toxin-positive *C. difficile* associated with severe diarrhea isolated in China discussed here belonged to clade 3, while all epidemic 027/ST1 and 078/ST11 strains were concentrated in clade 2 and clade 5, respectively (Fig. 1). Those results are in accordance with our previously reported phylogenetic tree generated using whole-genomic comparison [14]. What is more, the clade 3 branch also included another China—sourced toxigenic *C. difficile* strain ZJCDC-S82 which is also reported as a severe diarrhea-associated strain [44]. In addition, the other three recently-reported binary toxin-positive *C. difficile* (strains 103, 133, and 106) recovered from three ICU patients in China are also clade 3 strains [16]. These findings suggest that *C. difficile* clade 3 strains might contribute to the occurrence of CDI in China. In the phylogenetic tree, the same evolutionary branch includes *C. difficile* strains isolated from different places (Fig. 1), suggesting that there were no correlations between the bacterial genetic diversity and its geographic location. Meanwhile, even though all 027 or 078 strains were concentrated on the same clade, there were still strains sharing the same ribotype/sequence type being clustered in different clades (Fig. 1), suggesting that there was little correlation between the bacterial genetic diversity and its sequence type/ribotype. The phylogenetic analysis also showed that the clade 3 strains had a closer evolutionary relationship with the 027 strains that with the 078 strains (Fig. 1). Consistence with this, much less SNPs were identified between the ST201 strains in clade 3 against the 027 strain R20291 in clade than against the 078 strain M120 in clade 5 (Table 4). Besides, the average dN/dS of the ST201 strains against both the 027 strain and the 078 strain were significantly smaller than 1, suggesting a strong purifying selection during the evolutionary process [6].

The genomes of the binary toxin-positive ST201 strains as well as the epidemic 027 and 078 strains contained more than 40 antibiotic-resistance-related genes which confer the strains resistance to multiple antibiotics (Table 2). It has been proposed that the use of antibiotics is the most important risk factor for CDI [4], because *C. difficile* is resistant to multiple antibiotics that are commonly used for treating bacterial infections in clinical settings [2, 45]. Therefore, so many antibiotic resistance-related genes harbored in the ST201 strains may contribute to the bacterial pathogenesis. What is more, a large proportion (37.5%) of those antibiotic resistance genes were predicted to be associated with resistance to vancomycin, a kind of antibiotic commonly used for CDI treatment in clinic [46, 47]. Our result from antimicrobial susceptibility test demonstrated that strain LC693 is resistant to vancomycin, suggesting that those genes confer resistance of vancomycin to the strain. This might explain that enteral vancomycin is useless for treating the patient who is infected by strain LC693 [14].

Toxin expression is considered to be a key factor for the development of CDI [4], and PaLoc is responsible for encoding the clostridial toxins and regulating their expression [10]. Like the PaLoc reported in other clade 3 strains before [16, 33, 44], the PaLoc carried by the three ST201 strains discussed in this study as well as another clade 3 strain ZJCDC-S82 contained a mobile genetic element designated Tn*6218* inserted between *tcdE* and *tcdA* (Fig. 2). It is suggested that the insertion of Tn*6218* in PaLoc is clade-specific [16]. Consistence with this, this insertion element was not found in the PaLoc of R20291 in clade 2 and M120 in clade 5. In addition, the Tn*6218* in the three ST201 genomes were found to be flanked by two AT rich sequences. Previous studies suggested that those two AT rich sequences might have inserted into clade 3 PaLoc prior to the insertion of Tn*6218* and provide the insertion site of Tn*6218* [16]. For the other components of the PaLoc, it is worth to mention that although phylogenetic analysis using either MLST or whole genome comparison demonstrated a closer evolutionary relationship between the ST201 strains and the 027 strain R20291 (Fig. 1), the ST201 strains and the 078 strain M120 shared a more homologous *tcdA* and/or *tcdB* (Table 5). Further analysis needs to be performed to determine whether the toxin-yielding profile of the ST201 strain is closer to the 027 strain or to the 078 strain. For the toxin expression regulating genes, the *tcdC* gene is proposed to be a negative regulator for the toxin production, and the mutations within *tcdC* is observed to contribute to the toxin-production of some 027 strains [48, 49]. In our study, two main kinds of mutations were found within the *tcdC* gene ST201 carried by the ST201 strains compared to strain 630. The first one was an 18-bp deletion at positions 330–347 in the *tcdC*, and this mutation pattern was also found in the epidemic 027 strain R20291 (Fig. 3). However, this 18-bp in frame mutation has been found to have no effect on toxin production [50]. Instead, previous study reported that a deletion at position 117 in *tcdC* of the 027/ST1 strains compared to strain 630 resulted in the formation of a stop codon and truncation of the protein, and then caused increased toxin production further [51]. Even though this kind of mutation was not observed in the ST201 *tcdC* compared to the 630 *tcdC*, a nucleotide change occurred at position 185 (C → T) of the ST201 *tcdC*, which caused the formation of a stop codon here and therefore led to an early termination of translation as well as the disruption of the gene, may have a similar contribution to the toxin production of the ST201 strains.

Another factor contributing to the pathogenesis of the ST201 strains was the presence of the CdtLoc responsible for encoding the binary toxin in bacterial genomes. Previous data have found that the patients infected with *C. difficile* producing CDT had higher fatality rate (approximately 60%) than those infected with CDT-deficient strains [13]. A more recent study found that the binary toxin enhanced two PCR-ribotype 027 strains (R20291 and M7404) in mice by suppressing protective colonic eosinophilia [52]. Sequence comparisons demonstrated that the CdtLoc harbored in the ST201 strains was highly homologous to that of strain R20291, and the three genes *cdtA*, *ctdB* and *ctdR* carried by the ST201 CdtLoc were intact and also highly homologous to their corresponding genes harbored by R20291, respectively. These data suggest that the CdtLoc in the ST201 strain is active and the binary toxin encoded by it contributes to the pathogenesis of the ST201 strain. In particular, previous studies found that CdtR increased production of TcdA, TcdB and CDT in two epidemic 027 strains including R20291, but this regulation was not found in the 078 strain [53]. A R20291-*cdtR*-higly homologous *cdtR* identified in the ST201 strains may also have a similar role in positively regulating the production of the *C. difficile* toxins, and a truncated CdtR identified in most 078/ST11 strains may explain why the CdtR-mediated toxin regulation does not occur in the 078/ST11 strains [53]. In addition, whole genome sequence comparison identified a series of virulence-associated genes shared by the three ST201 genomes but not shared by both the R20291 genome and the M120 genome, the presence of these genes may also have a contribution to the bacterial pathogenesis.

## Conclusions

We summarized the genomic characterization of three binary toxin-positive ST201 strains in clade 3 in this study. While the presence of multiple fitness and virulence associated genes might form the pathogenesis basis of the binary toxin-positive ST201 strain, two main contents are likely to play the main role. (1) The presence of a number of antibiotic resistance associated genes in the strain especially the vancomycin resistant genes might increase the treatment difficulty of the bacterial infection; (2) the toxin producing required genes of the ST201 strain were highly homologous to the epidemic 027/ST1 strain; these genes might increase the virulence of the bacterium. Our work reveals the pathogenesis-basis of the ST201 binary toxin-positive strains in part. To our knowledge, this is the first time that the genomic characterization of the ST201 strains in clade 3 was discussed. As studies on clade 3 strains especially *C. difficle* ST201 are limited, the present study would have a contribution to understanding the pathogenesis basis of *C. difficle* ST201.

## Additional file

**Additional file 1: Table S1.** Genes identified to be specific for *Clostridium difficile* ST201 strains but absent in both 027/ST1 strain R20291 and 078/ST11 strain M120.

## Abbreviations

CDI: *Clostridium difficile* infection
CDS: coding DNA sequences
CDT: binary toxin
CdtLoc: binary toxin encoding locus
MLST: multilocus sequence typing
ORF: open reading frame
PaLoc: pathogenicity locus
SNP: single nucleotide polymorphism
